# Dominance in Domestic Dogs: A Quantitative Analysis of Its Behavioural Measures

**DOI:** 10.1371/journal.pone.0133978

**Published:** 2015-08-26

**Authors:** Joanne A. M. van der Borg, Matthijs B. H. Schilder, Claudia M. Vinke, Han de Vries

**Affiliations:** 1 Wageningen University Behavioural Ecology Group, Department of Animal Sciences, P.O. Box 338, 6700 AH, Wageningen, The Netherlands; 2 Utrecht University Faculty of Veterinary Medicine, Department of Animals in Science & Society, P.O. Box 80166, 3508 TD, Utrecht, The Netherlands; 3 Utrecht University Animal Ecology Group, Department of Biology, Padualaan 8, f3584 CH, Utrecht, The Netherlands; CNRS (National Center for Scientific Research), FRANCE

## Abstract

A dominance hierarchy is an important feature of the social organisation of group living animals. Although formal and/or agonistic dominance has been found in captive wolves and free-ranging dogs, applicability of the dominance concept in domestic dogs is highly debated, and quantitative data are scarce. Therefore, we investigated 7 body postures and 24 behaviours in a group of domestic dogs for their suitability as formal status indicators. The results showed that *high posture*, displayed in most dyadic relationships, and *muzzle bite*, displayed exclusively by the highest ranking dogs, qualified best as formal dominance indicators. The best formal submission indicator was *body tail wag*, covering most relationships, and two low postures, covering two-thirds of the relationships. In addition, both *mouth lick*, as included in Schenkel’s active submission, and *pass under head* qualified as formal submission indicators but were shown almost exclusively towards the highest ranking dogs. Furthermore, a status assessment based on changes in posture displays, i.e., lowering of posture (*LoP*) into *half-low*, *low*, *low-on-back* or *on-back*, was the best status indicator for most relationships as it showed good coverage (91% of the dyads), a nearly linear hierarchy (h’ = 0.94, p<0.003) and strong unidirectionality (DCI = 0.97). The associated steepness of 0.79 (p<0.0001) indicated a tolerant dominance style for this dog group. No significant correlations of rank with age or weight were found. Strong co-variation between *LoP*, *high posture*, and *body tail wag* justified the use of dominance as an intervening variable. Our results are in line with previous findings for captive wolves and free-ranging dogs, for formal dominance with strong linearity based on submission but not aggression. They indicate that the ethogram for dogs is best redefined by distinguishing body postures from behavioural activities. A good insight into dominance hierarchies and its indicators will be helpful in properly interpreting dog-dog relationships and diagnosing problem behaviour in dogs.

## Introduction

Generally stated, living in social groups can be beneficial for individual and species level survival for several reasons and in several circumstances, both in the short and long term (see [1]). Wolves and domestic dogs are *Canidea* species known for their high degree of sociality [2,3], but there is little quantitative data concerning the dynamics of their social organisation, dominance hierarchy and dominance style, affiliative relationships, coalition/alliance formationf and reconciliation behaviour. Such behaviour, if the social organisation in wolves can be taken as an example, appears to assist in coping with continuous change in several areas. For example, they contribute to hunting efficiency: prey size, availability of prey, prey detection, and to the care offered in a pack: learning opportunities, alloparental care; and territory defence [2,4]. At the same time, living in social groups may enhance competition for highly valued resources such as food and mates [5]. This competition can lead to conflicts that may compromise group stability and may even result in chronic stress and physical harm [6]. It can be alleviated by dominance hierarchies built from stable dyadic dominance relationships, allowing regulation of priority of access to highly valuable resources, and preventing fierce or recurring conflicts. The peacefulness of interactions within wild wolf packs impressed Mech, an experienced wolf observer [7]. Nevertheless, Mech’s remarks to this effect has led to a series of articles in the ongoing debate on whether or not dominance plays a role in the society of the wild wolf and consequently, whether dominance could constitute an important element in structuring social relationships between dogs and also between dogs and their owners (for review [8]). Unfortunately, in this debate the term dominance is widely used, often without reference to an underlying model or definition.

In our research, we rely on the model devised by van Hooff and Wensing [9], which has been applied to primates by de Waal [10] and used in many other studies of social living animals (free-ranging dogs: [11], bonobos: [12], macaque species: [13], wolves: [14], plains zebras: [15], Icelandic horses: [16], domestic pigs: [17]). The model runs as follows:

Members of a social group may differ in many aspects, including asymmetries regarding physical power, stamina, personality, weight, weaponry, age, and so on (see for detailed descriptions on criteria:[18]). These differences in personal properties are likely to influence the relationships between individuals (see also [19]) and may be stable for some time. Stable relationships between individuals may be correlated with more or less predictable differences in behaviours and predictable outcomes of conflicts. However, motivation may interfere and this may lead to some variation in the outcomes of conflicts over resources. It is thus only useful to speak of a dominance relationship between two individuals when a number of (behavioural) asymmetries correspond. Thus a number of different behaviours exchanged within each pair of animals should show corresponding main directions: e.g. individual A shows some relevant dominance related behaviours more frequently towards individual B than *vice versa*, and consequently some submission related behaviours consistent with these main directions could be shown more frequently by B towards A. If this is the case, then dominance may be regarded as a so called intervening variable that summarizes a set of behavioural differences between individuals [20,21]. If it then turns out that the individual group members can be ranked according to this intervening variable, the concept of dominance as defined above is applicable.

It should be made clear that animals do not need to have a notion of the concept of dominance in order to establish a dominance relationship and with it, a hierarchy. As computer simulations have shown, rank orders may arise automatically [22], when a few simple rules of giving or taking precedence are followed. Self-organisation is an underestimated aspect of social organisation in animal species. It arises through repeated encounters among group members, which are opportunities to gain information on the actual *strength* of opponents and help to avoid losing fights in the future [23]; thus learning plays a role in the formation of dominance relationships.

It’s important here to understand what dominance actually is. The definition of dominance by Drews [24] is analogous to the original definition by Schelderup-Ebbe [25]: the outcomes of agonistic dyadic interactions result in consistent winners being dominant and losers being subordinate. But dominance based on winning conflicts in agonistic contexts is not the only way to view it. Two more types of dominance, distinguished by primatologist de Waal [10], are based on either formal dominance or competitive ability. Formal dominance develops via the exchange of status information through ritualized and/or greeting signals that are independent of context. Competitive ability considers the motivation of animals to obtain or to possess resources. In canids, this has been measured using pairwise competition tests over bones or toys [26]. Competitive orders based on priority of access to food or water, however, are not necessarily in agreement with agonistic dominance [27] or formal dominance [28,11], although these are usually correlated.

The ritualized communication patterns that may serve a relative riskless establishment of dominance relationships (primates: [10], wolves: [29]) are of specific interest in the study on dominance and hierarchy formation. Such communication signals seem to deescalate conflicts and reduce the risk of physical injuries or worse [10]. A variety of ritualized signals, mostly body postures and facial expressions, were found to be shown in only one direction in dyadic encounters. These are described in primate species to serve as formal signals of dominance or submission (e.g. bare-teeth display in rhesus monkeys: [28]), and the same has been seen in wild wolves, captive wolves and free-ranging dogs [3,30–36]. This one-directional pattern is also seen in Van Hooff & Wensing [9], who were the first to study body postures as dominance indicators in a captive pack of wolves. Their findings showed that in a captive wolf pack, two postural displays (namely high posture and low posture), accompanied by seven agonistic and affiliate behaviours, were displayed in the dyads in mainly one direction and were therefore better indicators of formal dominance than the seven behaviours *per se*. On the basis of these two postures, it was possible to construct a rank order that was not only transitive but also linear (Landau’s linearity index h>0.9). In this way, formal dominance was proven and the significant correspondence between the rank orders constructed on the basis of several formal status indicators justified the application of the dominance concept in a captive wolf pack.

A different set of indicators was used by Bradshaw and co-workers [37] in their unpublished qualitative study in which they examined a semi-permanent all-male group of 19 neutered domestic dogs, searching for a dominance hierarchy based on “confident” (e.g. *growl*, *inhibited bite*, *stand over*, *stare at*, *chase*, *bark at*, *mount*) and “submissive” (e.g. *crouch*, *avoid*, *displacement lick/yawn*, *run away*) behaviours. These behaviours were presumed to be useful as rank indicators but not investigated for their usefulness, as Van Hooff & Wensing had done in their captive wolf study [9]. Since they found no linear hierarchy, Bradshaw et al. [37] concluded that dominance does not play a role in the domestic dog and that dogs do not strive for a dominant position. Their study excluded posture as a behavioural variable previously shown to be an important variable for dominance relationship assessment in wolves [9].

Dominance hierarchies based on aggression and submission were found in two packs of Indian free-ranging dogs [38], but the properties linearity, directional consistency and coverage were not assessed. A later recalculation of these properties for these behaviours ([39], p.78), showed high levels of linearity in the two packs. Linearity is also shown in a study on Italian free-ranging dogs [11] in which three types of dominance were investigated and compared: the formal dominance, agonistic dominance and competitive ability [10]. In that study, presumed behaviours belonged to one of the three clusters (aggressive, dominance and submissive behaviour) and these clusters were investigated to determine whether they could be used to fit dogs into a linear dominance hierarchy. The findings showed that (1) the agonistic-dominance hierarchy was substantially linear, (2) the submissive-affiliative patterns fulfilled the criteria of formal submission signals (but were not observed among all dog pairs) and (3) the competitive rank order (based on gaining access to food) was predicted reasonably well by the agonistic rank order.

So far, nearly linear hierarchies constructed from systematic, quantitative data on submissive (but *not* agonistic) behavioural measures, appear in one study on captive wolves [9] and in two studies on free-ranging dogs [11,39] but not in group housed domestic dogs [37]. These contradictory results concerning the role of dominance in canids have led us to investigate this aspect of social organisation in a group of domestic dogs in more depth. We hypothesize that the dominance model as sketched above is applicable in our group of domestic dogs, just as in captive wolves [9] and in free-ranging dogs [11,39]. More specifically, we expect that postural communication and submissive behaviours play a major role in status communication, since these have been shown to be the best indicators in wolves and free-ranging dogs. Since wolves and dogs are genetically very close [40,41], despite domestication we expect to find the same social organisation, meaning a (nearly) linear hierarchy based on formal dominance with stable relationships.

In the present research, we firstly address the question of the usefulness of postural and behavioural variables as status indicator in domestic dogs. To this end, we strictly distinguished postures (including ear, tail, and body positions) from all the other behavioural variables (e.g. *bark*, *growl* or *pilo-erection*). Subsequently, we compared these behaviours and postures with respect to their qualities as indicators of dominance or submission in dyadic relationships and characterised the constructed rank orders in terms of linearity. As an additional element, we investigated the steepness of the one rank order that stood out in terms of linearity, transitivity and coverage [42,43]. Steepness provides a measure of the strength of the asymmetry between neighbouring ranked individuals concerning a relevant behavioural measure for instance, the summed dyadic proportions with which a group member receives submissive acts or wins dyadic encounters indicates overall individual dominance success. Finally, cluster analysis on a subset of postures and behaviours was used to reveal clusters possibly indicative of different aspects of dominance and aggression that would justify the use of the concept of dominance as an intervening (= summarizing) variable [9,20].

## Materials and Methods

### Ethics Statement

This study complies with Dutch regulations regarding the ethical treatment of laboratory domestic dogs. Research permission to conduct the study was granted by the Faculty of Veterinary Medicine and the Faculty of Biology of the Utrecht University. Research protocol aimed at self-regulation of conflicts in the dog group. Serious fights that could possibly inflict wounds were prevented by human interventions, in some cases even leading to removal of the aggressor from the group.

### Study group and housing

In this study, we reanalysed data originally gathered from a newly formed group of domestic dogs at the dog kennel of the Faculty of Veterinary Medicine of the Utrecht University, the Netherlands. The group consisted in total of sixteen dogs of different breeds and age: three adults, two sub adults, seven juveniles (of which four were litter mates) and three pups. Their individual features such as breed, gender, age and weight are listed in Table 1. All dogs were sexually intact. Of these 16 dogs, ten (referred to as the *core group*) were present during the total observation period of 12 weeks. In week 5, three of the 13 original dogs were removed from the group: A (subadult female) and F (adult female) were removed due to a severe fight, and J (adult female) was removed due to advanced pregnancy. In week 10 of the observation period, three pups (G, R, Y) were introduced into the group. With exception of these pups, the dogs were housed individually in kennels outside observation hours.

**Table 1:** The composition of the dog group by name, code, breed, sex, age and weight.

	Name	Code	Breed	Sex	Age^1^ (months)	Weight^1^ (kg)
**Adults**	Flets	F	Beagle	F	78	10.2
(>18 months)	Juultje	J	Beagle	F	44	11.5
	Issie	I ^2^	Cairn terrier	F	24	6.3
**Subadults**	Pasha	P ^2^	Malinois	F	12.5	25.3
(9 months to 18 months)	Astarte	A	Great Dane	F	9.3	44.9
**Juveniles**	Vlek	V ^2^ ^3^	Beagle	M	6.7	11.0
(3 months to 9 months)	Zwart	Z ^2^ ^3^	Beagle	F	6.7	11.9
	Streep	S ^2^ ^3^	Beagle	F	6.7	10.2
	Kraag	K ^2^ ^3^	Beagle	F	6.7	9.9
	Witband	W ^2^ ^4^	Beagle	M	5.7	10.6
	Tanja	T ^2^	Doberman Pinscher	F	5	16.4
	Umpie	U ^2^	Dalmatian	F	4.8	16.1
	Bodo	B ^2^	Labrador Retriever	F	3.4	14.5
**Pups**	Geel	G	German Shepherd	F	2.5	10.3
(< 3 months)	Reu	R	Dutch Shepherd	M	2.2	8.7
	Yazzoo	Y	German Pointer	F	2	6.2

^1^ = at the start of the introduction in the group

^2^ = core group of 10 dogs during the total observation period of 12 weeks

^3^ = littermates; offspring of adult female Flets

^4^ = son of adult female Juultje

F = female

M = male

The indoor dog kennels were connected by a corridor to an outdoor enclosure of 273 m^2^ (10.5 m. x 26 m.) covered with low undergrowth vegetation, grass and sand. On observation days (Monday through Friday), the dogs were brought into the outdoor enclosure at dawn (5–6 am). At the end of the observations the dogs were brought back to their own kennels.

The dogs were fed regular dog food pellets twice a day: in the morning by the observers at three fixed feeding places in the outdoor enclosure and in the afternoon by the dog caretaker in their own kennels. Some old bones and a white plastic platform (height 35 cm with 1m by 0.5m) were available to play with in the outdoor enclosure. Drinking water was available ad libitum from a drinking trough.

During the weekends, dogs were regularly taken home by students for socialisation.

### Data collection

Preliminary observations during a two months observation period were carried out in order 1) to describe the ethogram and learn the codes for the behavioural variables, and 2) to practise the observation and recording methods until more than 90% agreement among the two observers was reached during live registrations. Data collected during this period were excluded from analyses. Systematic observations by two observers started two months after the formation of the group and were conducted from 11 June until 31 August 1984, Mondays through Fridays, totalling 60 observation days spread over 12 weeks.

Quantitative recording was conducted during a total of 323 hours. Of these, 107 hours of the last four weeks were used for analyses after relationships were considered to be stabilized: 58 hours focal animal sampling and 49 hours *ad libitum* sampling [44]. Observers used both methods equally often, changing methods between them on a daily basis. The unit of focal animal time per dog was 10 minutes. Every dog in the pack was sampled twice daily following a fixed observation order, starting each day with the dog next in the order. The time between two consecutive observations on the same animal depended on the number of animals in the group. *Ad libitum* sampling was focussed on play and agonistic interactions, specifically between dogs that seldom interacted.

### Behaviour variables

As in wolves, different body postures in dogs can be assessed on the basis of specific combinations of postural components: position of the tail and head, the ears and the bending of the hind legs and the straightness of the back [30,34]. In the studies on wolves [9,14] three distinct postures were used: *high posture* (head up and tail upright, ears pricked, straight back and straight legs), *low posture* (head low, tail down or tugged between the legs and somewhat bent, and ears folded backwards) and *neutral posture* (as an intermediate posture). By adding four additional postures, we employed a more detailed postural ethogram distinguishing in total 7 postures (Table 2). The postures *half-high* and *half-low* were added to capture intermediate positions. The postures *neutral* and *low* were scored, if performed in a dorsal to lateral lying position, as *back* and *low-on-back*. Posturing was scored relative to breed, because breed affects posture, e.g. a *neutral* posture for a Beagle looks different from that of a Labrador retriever.

**Table 2:** Ethogram for tail and ear positions for 7 postures.

	Positions	
Posture^1^	Tail	Ears
**High**	maximum highest carriage	maximally erected (standing) or held forward (hanging)
**Half-high**	partially highest carriage and held above the horizontal line of the back	partly erected or hanging forward, higher than Neutral
**Neutral**	follows line of hind quarter and held around the horizontal line of the back	held relaxed, partly sideward
**Back**	as in Neutral but in a dorsal or lateral lying position	as in Neutral
**Half-low**	lower than Neutral but not held against or between the hind-legs	partly retracted into the neck, lower than Neutral
**Low**	the upper side of tail against hind quarter and s-shaped, or lower tugged between the hind-legs	maximally retracted into the neck (standing) or held backwards (hanging)
**Low-on-back**	as in Low but in a dorsal or lateral lying position	as in Low

^1^The range of tail carriage (from high to low) differs strongly between breeds and have been taken into account in assessing the posture.

Postures were consistently recorded first and then the accompanying behaviour elements were recorded. Thus descriptions of postures excluded behaviours, with the exceptions of *body tail wag* and *pass under head*; tail position was also included in the definition to characterize behaviours accurately. From the original 75 elements in the ethogram, 24 elements were researched for their suitability as status indicator. Their inclusion was based on their correspondence with behaviours described in the ethograms for captive wolves and free-ranging dogs, and on the traditional presuppositions in the domestic dog literature that they are related to dominance or subordination [37,45].

### Recording of dyadic interactions

While three types of interactions were recorded (dyadic, triadic and polyadic), to assess the usefulness of dominance or subordinate status indicators we analysed only dyadic interactions, which followed the basic format: Actor—Posture and Behaviour(s)—Recipient—Posture and Behaviour(s).

For an assessment of status in dyadic relationships, the observers also compared the beginning and ending of interactions, for changes in posture displays. This was done directly after the dogs ended their interaction, in the following three contexts:

1. Spontaneous: a dog spontaneously lowered its posture into a lower posture as shown previously, namely one of the low postures (= *half-low*, *low* or *low-on-back*) or *on-back*, towards a recipient that showed no signs of aggression.
2. Agonistic context: in reaction to opponent’s aggressive behaviour (*stare*, *pilo-erection*, *growl*, *show teeth*, *snap*, *lunge*, *bite*, *muzzle bite*, *fight*, *stand over*, *chase*, *bark*) a dog lowered its posture into one of the low postures or *on-back*, or, if the dog was already showing one of the low postures or *on-back*, the dog started to signal behaviours indicative of fear, submission or avoidance (*tongue flick*, *freeze*, *look away*, *body tail wag*, *lick mouth*, *pass under head*, *high pitch vocalisations*, *flee*, *retreat*, *shrink back*).
3. Competitive context: a dog lowered its posture into one of the low postures or *on-back* towards an opponent that tried to take or took an object or bone, or pushed the dog away from a feeding place or burying site, or the dog controlling the resource showed a *neutral* or *higher* posture.

A change in posture display as described above by one of the interactors was labelled *Lowering of Posture* (= *LoP*).

### Statistical analysis

Observational data gathered by the focal animal sampling method were directly typed into a handheld computer. *Ad libitum* sampled data were noted on special check sheets and typed into a computer that same day.

Based on preliminary analysis of dominance and the development of the rank order, we decided to split the observation period in three periods of 4 weeks. All behavioural variables were regarded as point events and frequencies were calculated per period of 4 weeks. For analysis of dominance only dyadic interactions of the last 4 weeks were used, because relationships appeared to be stable during this period.

The extent to which the postures and behaviours are suitable indicators of dominance was assessed by calculating three properties for each of the seven postures and 24 behaviours: linearity, directional consistency and coverage, using MatMan 1.1 (Noldus technology, Wageningen, the Netherlands). For comparison with earlier studies on canids, we used the improved linearity index (h’) [46], and determined the rank order most consistent with a linear hierarchy via the I&SI method [47]. Linearity is considered to be strong with a linearity index of 0.9 or higher [48]. The directional consistency index is Σ(*H*_*ij*_-*L*_*ij*_)/Σ(*H*_*ij*_+*L*_*ij*_), where summation is across all dyads *i*,*j* (*i*>*j*), and *H*_*ij*_ is the highest frequency count and *L*_*ij*_ the lowest frequency count within dyad *i*,*j* [9,12]. It is a measure of the overall within-dyad unidirectionality (0 indicates complete symmetry, 1 indicates complete unidirectionality). Coverage is the proportion of non-zero dyads in the matrix [9]. All matrices with submission related variables were transposed, i.e. mirrored around the diagonal.

Calculation of the steepness of a rank order was based on the David’s score (DS) [49], considered to be a suitable measure of overall individual dominance success which takes the relative strength of other individuals into account [43,50]. Subsequently, we converted the DS into normalized DS (NormDS), based on *P*_*ij*_, i.e. the dyadic proportions of received changes in posture display (based on the exchanged *LoP* frequencies in dyads). The NormDS values were plotted against the ordinal ranks of these NormDS values (with equal NormDS values assigned different ranks) [42]. With the statistical R package ‘*steepness’* [51] ordinary least-squares linear regression was used to find the best-fitting straight line and the slope of this line was used as a measure of the steepness of the dominance hierarchy [42]. With a randomization test procedure (20,000 randomizations) the significance of the right-tailed P value was obtained by calculating the proportion of times that a randomly generated steepness under the null hypothesis is greater than or equal to the actually observed steepness.

Correlation between the *LoP* rank order based on NormDS (using *P*_*ij*_; for comparison with cluster analysis using NormDS based on D_ij_ see S1 File) and the individual factors age and weight was tested with Spearman rank correlation.

To identify behavioural variables that co-vary, a cluster analysis with average linkage clustering method and the Pearson product moment correlation as similarity measure was performed on the NormDS based on *P*_*ij*_ of 2 postures and 11 behaviour variables, that were selected for having significant *h’* and less than 25% blank relationships [52].

## Results

### Body postures as suitable status indicator

The 7 postures (*N* = 8,873 observations) recorded for the 10 core group dogs were examined for linearity, directional consistency and coverage (Table 3). *High posture* had strong linearity (h’ = 0.93), was highly unidirectional (DCI = 0.90), covered most relationships (84.4%) and therefore qualified best as status indicator for dominance. *Low* and *low-on-back* did not show significant linearity due to low coverage (53.3% and 37.8% respectively), but were highly unidirectional (DCI>0.95). Combined, these two low postures (*N* = 186) showed significant linearity (h’ = 0.71) and very high unidirectionality (DCI = 0.99); consequently the coverage increased to 66.6%. A low posture, whether shown in a standing (*low*) or a lying position (*low-on-back*), qualified as best formal status indicator for dyadic relationships. Although the postures *half-high*, *neutral* and *half-low* all showed significant linearity and nearly 100% coverage, they were not good indicators because they were shown in 75% or more relationships in both directions (DCI = 0.40, 0.38 and 0.76, respectively).

**Table 3:** Properties of 7 postures: frequency (*N*), improved linearity index (h’), directional consistency index (DCI), coverage (Unknown), unidirectionality (1-Way), bidirectionality (2-Way) and number of ties (Tied), over the last 4 weeks of observations.

	*N*	h' ^1^	DCI ^2^	Unknown ^3^		1-Way ^4^		2-Way ^5^		Tied ^6^	
**High**	249	**0.93** *(p = 0.0001)	**0.90**	7	(15.6%)	28	(62.2%)	10	(22.2%)	2	(4.4%)
**Half-high**	3 904	0.85(p = 0.0002)	0.40	0	(0%)	0	(0%)	45	(100%)	0	(0%)
**Neutral**	2 814	0.63(p = 0.006)	0.38	2	(4.4%)	9	(20.0%)	34	(75.6%)	4	(8.9%)
**Half-low**	1 616	**0.95**(p = 0.0001)	0.76	0	(0%)	9	(20.0%)	36	(80.0%)	0	(0%)
**Low**	132	0.61(p = 0.03)	**1**	21	(46.7%)	24	(53.3%)	0	(0%)	0	(0%)
**Low-on-back**	54	0.38(p = 0.3)	**0.96**	28	(62.2%)	16	(35.6%)	1	(2.2%)	0	(0%)
**On-back**	104	0.38(p = 0.3)	0.79	20	(44.4%)	17	(37.8%)	8	(17.8%)	1	(2.2%)

^1^Improved Linearity index [46]

^2^Directional consistency index [9]

^3^Number and percentage of unknown relationships

^4^Number and percentage of one-way relationships

^5^Number and percentage of two-way relationships

^6^Number and percentage of tied relationships

*Indexes ≥ 0.9 are in bold.

The assessment of status within dyadic relationships based on *LoP* display (Table 4) revealed strong linearity (h’ = 0.94, p<0.0001), high unidirectionality (DCI = 0.97) and high coverage (91%). Lowering of posture during an interaction was thus the status indicator best suited to assessing dominance in this group of domestic dogs.

**Table 4:** Properties of Lowering of Posture (LoP): frequency (*N*), improved Landau’s linearity (h’ index), direction consistency (DCI), coverage (Unknown), unidirectionality (1-Way), bidirectionality (2-Way) and number of ties (Tied), over the last 4 weeks of observations.

	*N*	h' ^1^	DCI ^2^	Unknown ^3^		1-Way ^4^		2-Way ^5^		Tied ^6^	
**LoP**	552	**0.94** *(p = 0.0001)	**0.97**	4	(8.9%)	34	(75.6%)	7	(15.6%)	3	(6.7%)

(^1^ to ^6^: see legend below Table 3)

*Indexes of ≥ 0.9 are in bold.

### Behaviours as suitable status indicator

A selection of 24 behaviours (for ethogram see Table 5) were included in our analysis, assuming to reflect sufficiently the pack’s behavioural repertoire related more or less to dominance and subordinate behaviour. Table 6 shows the linearities, directional consistencies and coverages of these 24 behaviours.

**Table 5:** Ethogram for 24 behaviours in dogs (adapted from Zimen [34] and van Hooff and Wensing [9]).

*Behaviour element*	*Description*
Mouth lick	Licking repeatedly with fast movements directed to the recipient’s mouth corners
Body tail wag	Accelerated, irregular movement of the tail, often also the hindquarter is moving, in a neutral or lower posture (posture is included to distinguish from normal tail wag, see below)
Pass under head	Passing from the lateral side closely underneath the head of the recipient, often short nose-chin contact with the recipient, in a neutral or lower posture
Stare	Intense fixating look towards recipient with tensed body, for a minimal duration of 2 seconds
Pilo-erection	Raising the hair on one or more upper parts of the body (neck, shoulder, hindquarter) and/or tail base
Growl	Low-pitched rumbling, fairly monosyllabic vocalization from the dog’s throat
Show teeth	Baring of the teeth, which become partly or totally visible.
Snap	Attempt to bite while moving not more than 1 or 2 steps (about ½ meter) in the direction of the recipient, without physical contact
Lunge	Attempt to bite while moving over a distance between ½ to 3 meters in the direction of the recipient, without physical contact
Bite	Taking any part of the recipient’s body between the jaws with sufficient pressure that could cause harm to the recipient
Fight	Severe, offensive aggressive interaction between two dogs, including aggressive elements like lunge and bite
Shrink back	Accelerated movement directed away from the recipient over a distance up to 1 meter
Retreat	Accelerated movement directed away from the recipient over a distance from 1 to 3 meters
Flee	Running away from the recipient over a distance of 3 meters or more, with head in opposite direction of the recipient
Stand over	Standing over the recipient’s body, with four paws on the ground, in a neutral or higher posture
Muzzle bite	Inhibited biting over the recipient’s snout from above or from the side
Tongue flick	Showing one or more brief licking movements with tongue directed towards nose and head oriented towards recipient, without physical contact
Look away	Turning only the head away from the recipient, while staying on the same spot
Freeze	General rigidity of the body, with exception of the tail, and no staring towards the recipient
Approach	In normal pace walking (not accelerated) towards the recipient up to a distance of 1 meter or less
Take away object	Taking away object or bone that is in possession of the recipient
Bark	Loud and repetitive barking (characteristic for dogs) directed towards the recipient
Tail wag	Non accelerated, regular sideward movements of the tail, about in one plane
Paw on	Placing one or both front paws on the recipient’s head or back

**Table 6:** Properties of 24 behavioural elements: frequencies (*N*), improved Landau’s linearity (h’ index), direction consistency (DCI), coverage (Unknown), unidirectionality (1-Way), bidirectionality (2-Way) and number of ties (Tied), over the last 4 weeks of observations.

	*N*	h' index ^1^	DCI ^2^	Unknown ^3^		1-Way ^4^		2-Way ^5^		Tied ^6^	
Mouth lick	193	0.4(p = 0.18)	**0.97** *	25	(55.6%)	19	(42.2%)	1	(2.2%)	0	(0%)
Body tail wag	316	0.82(p = 0.0003)	**0.96**	11	(24.4%)	30	(64.4%)	5	(11.1%)	1	(2.2%)
Pass under head	142	0.45(p = 0.16)	**1**	29	(64.4%)	16	(35.6%)	0	(0%)	0	(0%)
Stare	996	0.85(p = 0.0002)	0.50	1	(2.2%)	7	(15.6%)	37	(82.2%)	1	(2.2%)
Pilo-erection	295	0.66(p = 0.009)	0.68	10	(22.2%)	15	(33.3%)	20	(44.4%)	1	(2.2%)
Growl	608	0.52(p = 0.04)	0.70	1	(2.2%)	17	(37.8%)	27	(60%)	3	(6.7%)
Show teeth	337	0.55(p = 0.03)	0.74	9	(20.0%)	22	(48.9%)	14	(31.1%)	2	(4.4%)
Snap	231	0.57(p = 0.03)	0.74	10	(22.2%)	22	(48.9%)	13	(28.9%)	1	(2.2%)
Lunge	124	0.59(p = 0.026)	**0.94**	18	(40.0%)	24	(53.3%)	3	(6.7%)	1	(2.2%)
Bite	28	0.29(p = 0.49)	0,43	33	(73.3%)	7	(15.6%)	5	(11,1%)	3	(6.7%)
Fight	18	0.24(p = 0.60)	0.11	38	(84.4%)	2	(4.4%)	5	(11.1%)	5	(11.1%)
Shrink back	155	0.63(p = 0.01)	0.66	10	(22.2%)	18	(40.0%)	17	(37.8%)	4	(8.9%)
Retreat	116	0.50(p = 0.07)	0.76	12	(26.7%)	22	(48.9%)	11	(24.4%)	6	(13.3%)
Flee	19	0.38(p = 0.27)	**0.98**	30	(66.7%)	14	(31.1%)	1	(2.2%)	1	(2.2%)
Stand over	18	0.32(p = 0.41)	**1**	36	(80.0%)	9	(20.0%)	0	(0%)	0	(0%)
Muzzle bite	101	0.33(p = 0.40)	**0.98**	35	(77.8%)	9	(20.0%)	1	(2.2%)	0	(0%)
Tongue flick	557	0.83(p = 0.0001)	0.72	1	(2.2%)	17	(37.8%)	27	(60.0%)	4	(8.9%)
Look away	989	**0.92**(p = 0,0001)	0.78	0	(0%)	12	(26.7%)	33	(73.3%)	1	(2.2%)
Freeze	1 198	**0.94**(p = 0.0001)	0.78	0	(0%)	14	(31.1%)	31	(68.9%)	2	(4.4%)
Approach	1 744	0.47(p = 0.05)	0.35	0	(0%)	0	(0%)	45	(100%)	1	(2.2%)
Take away object	123	0.53(p = 0.05)	0.61	18	(40.0%)	18	(40.0%)	9	(20.0%)	1	(2.2%)
Bark	361	0.43(p = 0.14)	0.49	0	(24.4%)	18	(40.0%)	16	(35.6%)	4	(8.9%)
Tail wag	507	0.86(p = 0.0001)	0.55	0	(0%)	11	(24.4%)	34	(75.6%)	4	(8.9%)
Paw on	292	0.34(p = 0.29)	0.53	13	(28.9%)	12	(26.7%)	20	(44.4%)	6	(13.3%)

(^1^ to ^6^: see legend below Table 3)

* Indexes ≥ 0.9 are in bold

*Body tail wag* was the most reliable status indicator of subordination for most dyadic relationships, with strong linearity (h’ = 0.82), high unidirectionality (DCI = 0.96) and good coverage (75.6%). Neither *mouth lick* nor *pass under head* showed significant linearity, due to low coverage (44.4% and 35.6% respectively), but both were highly unidirectional (DCI ≥ 0.97) and therefore both qualified as indicators of subordination for some dyadic relationships. Combining these two behaviours (comparable to active submission as described by [9,11,31]) did little to increase the values of the properties. Further inspection of matrices revealed that these two behaviours were almost exclusively shown towards the male W and female P (138 out of 142 and 181 out of 193, respectively).

Five aggressive behaviours (*stare*, *pilo-erection*, *growl*, *show teeth*, *snap*) were highly bidirectional (0.50 ≤ DCI ≤ 0.74), but did show significant linearity (p ≤ 0.04). Interestingly, with increasing intensity of aggression, unidirectionality increased, with the more intense aggressive behaviour *lunge* showing the highest unidirectionality (DCI = 0.94). Inspection of this matrix revealed that *lunge* was almost exclusively shown towards the two females T and U (106 of 124) by lower ranking dogs showing lower postures than their receivers, indicating this type of aggression is likely to be protest or fear motivated.

The most intense aggressive behaviours *bite* and *fight* had low frequencies (28 and 18, respectively) indicating low levels of extreme aggression (wounds inflicted) in this group after exclusion of the one pair that fought. Because of low unidirectionalities (DCI = 0.43 and 0.11, respectively), these two behaviours are not suitable as status indicators. The most intense fear behaviour *flee* was highly unidirectional (DCI = 0.98), but had very low frequency (*N* = 19).

*Stand over* and *muzzle bite*, both assumed to be related to dominance, revealed no significant linearity (h’ = 0.32 and h’ = 0.33, respectively) due to low coverage (20% and 22.2%, respectively). Besides *Stand over*, with the highest unidirectionality (DCI = 1), had very low frequency (*N* = 18), and therefore was disqualified as a status indicator. By showing very high unidirectionality (DCI = 0.98) and sufficient frequency (*N* = 101) *muzzle bite* qualified as a useful status indicator of dominance for some relationships. Inspection of this matrix revealed that *muzzle bite* was exclusively shown by the three highest ranking dogs, but mostly by female P (87 of 101).

The behaviours *tongue flick*, *look-away* and *freeze* showed significant and high linearity (h’>0.80), but due to insufficiently strong unidirectionality (DCI < 0.80) were not useful as status indicators. Miscellaneous behaviours such as *take away object*, *bark*, *paw on*, *approach* and *tail wag* were not useful as status indicators due to insufficient coverage and/or low directionality.

### Steepness of *LoP* rank order

**Fig 1:**
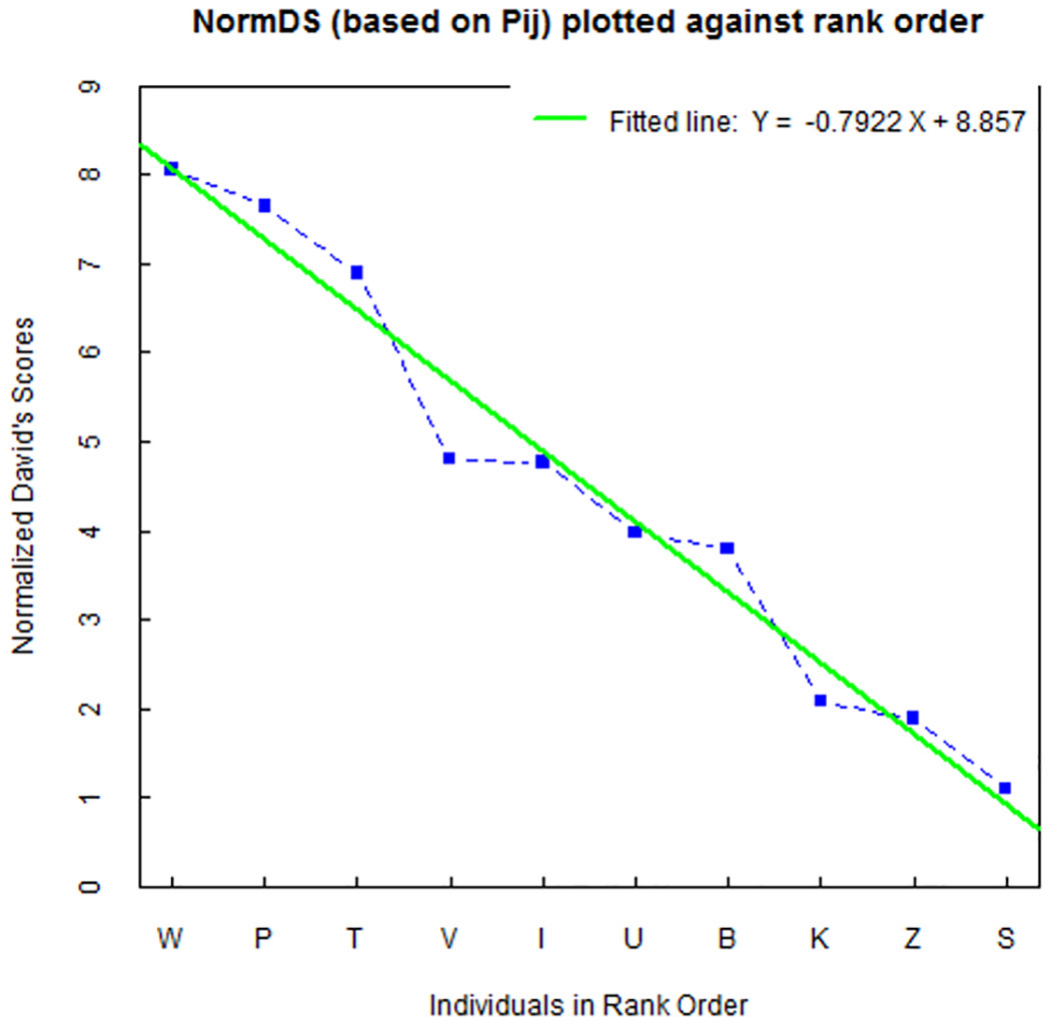
Steepness of rank order. The normalized David’s scores (NormDS based on *P*_*ij*_) for the *LoP* matrix plotted against the rank of 10 dogs, ranked from dog W (highest NormDS = rank 1) to dog S (lowest NormDS = rank 10). The steepness of this rank order (i.e. the absolute value of the slope of the fitted line) is 0.79.

As *LoP* qualified best for ordering the dogs into a formal linear hierarchy, we assessed the steepness of this rank order (Fig 1) based on the normalized DS values of the *LoP* matrix (S1 Appendix). The steepness of the observed matrix is 0.79, which differs significantly (right-tailed *P* = 0.0001) from the steepness value 0.32 expected under the null hypothesis.

The hierarchy in this group of dogs turned out to consist of three layers: first the highest ranking male W and two females P, T with NormDS values between 8 and 7, then a group of four middle ranking dogs V, I, U, B with NormDS values between 5 and 4, and finally a group of three lowest ranking dogs K, Z, S with NormDS values between 2 and 1. Most dyadic relationships in the lowest ranking subgroup (females K, Z, S; all from the same litter) remained unresolved since we hardly observed *LoP*, *high posture* or *body tail wag*, although they approached each other often (except dogs Z and S) (see matrices in the S1 Appendix).

### Correlation between rank and individual factors

No significant correlation was found between rank order (based on the NormDS of *LoP*) and weight (Spearman rank correlation: *r*_*S*_ = 0.46, *N* = 10, *P* = 0.09) or age (Spearman rank correlation: *r*_*S*_ = 0.14, *N* = 10, *P* = 0.35). With regard to sex and rank, no statistical conclusion could be drawn, because only two males were in the group.

### Co-variation between behaviour variables

To assess the usefulness of dominance as an intervening variable, we analyzed the co-variation between the variables that met the criteria of 25% coverage and significant linearity: postural variables *LoP*, *high posture*, and 11 behaviours. The cluster analysis of the Pearson correlation matrix of these variables revealed three clearly distinct clusters that strongly co-varied and some loosely linked behaviours (S1 Fig).

In the first cluster, the behaviours *freeze*, *tongue flick*, *look away* and *show teeth* strongly corresponded. These behaviours indicated either conflict (*tongue flick* and *look away*) or agonism (*freeze* and *showing teeth*). We labelled this group Agonistic-conflict behaviours. The second cluster included the behaviours *body tail wag* and *high posture*, as well as *lowering of posture* performed in the three different contexts (matrices shown in S1 Appendix) that qualified as best status indicators. The NormDS values of these postures and behaviour correlated strongly: *LoP* with *high posture* (*r* = 0.84, *N* = 10, P<0.001) and with *body tail wag* (*r* = 0.92, *N* = 10, P<0.001), and *body tail wag* with *high posture* (*r* = 0.84, *N* = 10, P<0.001). Following van Hooff and Wensing [9] we labelled this group dominance-subordination status signals. The three aggression related behaviours, *growl*, *snap* and *pilo-erection*, clearly clustered apart from the other two clusters. Finally, *tail wag* loosely linked to this Aggression cluster, while *stare* and *shrink back* were loosely linked to the two aforementioned clusters.

## Discussion

This study in a group of domestic dogs is the first that quantitatively researched postural and behavioural measures in dyads for their suitability as formal status indicator. We found one posture (*high posture*) and one behaviour (*muzzle bite*) suitable as formal signals for expressing dominance. Furthermore, two low postures (*low* and *low-on-back*) and three behaviours (*body tail wag*, *mouth lick* and *pass under head*) were found to be formal status signals of submission. However, only *high posture* and *body tail wag* covered most relationships. The assessment based on the change in postural display (*lowering of posture*, LoP) was the best formal indicator for expressing submission. The high correlation between the rank orders for *LoP*, *body tail wag* and *high posture* justifies the use of the term dominance as the intervening variable in domestic dogs. Moreover, for the first time, steepness [42] has proven its usefulness when describing the social organisation of a group of domestic dogs in terms of dominance style [10].

### Status indicators

Postural display is an important feature in intraspecific relationships in wolves. As predicted, we found the postural display *high posture* to be suitable in our dog group as a formal status signal for dominance and the two low postures *low* and *low-on-back* to signal submission. This is in line with the quantitative findings for captive wolves [9], in whom both dominant and subordinate roles are signaled by postural displays that might be considered a form of metacommunication [9]. Moreover, we found our determination of dominance relationships was reliable for most group members when we assessed the change of postural display during dyadic interactions, i.e. *lowering of posture* (*LoP*): changing to a lower posture (e.g. *half-low*, *low*, *low-on-back*, *on-back*) during an interaction. *LoP* was the best variable for determining linear rank order (linearity h’ = 0.94): *lowering of posture* can be seen as context-independent, ritualized postural signaling expressing a subordinate status.

In addition to postural displays, we also found certain behaviours to be suitable as status indicators. The strongest of these appears to be *body tail wag*, representing a ritualized signaling of acceptance of subordinate status and at the same time communicating friendly intentions. This *body tail wag*, along with *mouth lick* and *pass under head*, included in Schenkel’s description of active submission [31], stand out in our study group as formal status indicators of subordination. *Mouth lick* and *pass under head* did not occur in all relationships in our study, but were almost exclusively received by the highest ranking male and female. This finding strongly corresponds to what has been observed in free-ranging dogs, where *mouth lick* and “*tail wagging with low tail*” (comparable to *body tail wag*) were found to be formal submission signals not covering all relationships [11]; the same was also seen in the study on 24 domestic dogs in a daycare center, where muzzle licking behaviour was found to meet most dominance criteria but not to cover all relationships [53]. We stress that analyzing separately the constituent elements of active submission (*mouth lick*, *body tail wag and pass under head*) enabled us to reveal differentiation regarding the display of these status indicators in the social organisation of domestic dogs.

In descriptive wolf studies, the behaviour *muzzle bite* is claimed to be useful as an indicator of dominance shown by the breeding pair or parents forcing offspring to the ground [31,54], although a quantitative analysis of this behaviour in a captive wolf group did not reveal this behaviour to be a reliable status indicator [9]. Commonly it is assumed that dogs lost this dominance assertion behaviour during domestication [34,55]. However, in our mix breed dog group, *muzzle bite* behaviour was shown most frequently by the highest ranking female P towards the middle ranking female dogs T, U and B. Also the highest ranking male W displayed this specific behaviour towards the middle ranking females (U and B). Our analysis showed *muzzle bite* to be a formal signal of dominance (cf [56]), and moreover, we may even categorize this behaviour as an exclusive status signaller, as it was only employed by the highest ranking male and female towards lower ranking dogs and has also been described in family groups of wild wolves [54].

Most wolf researchers indicated the tail posture to be most indicative for assessing dominance (e.g. [30,31,35,57]), with the first quantitative evaluation of the tail posture in wolves done by Van Hooff and Wensing [9]. This metacommunication by means of postural displays occurs in all contexts. As every behaviour is accompanied by a posture, postures add an extra signal to the behaviour corresponding the relative status of the individuals involved. Therefore, as an example, to identify dominance related forms of aggression from other forms of aggression, the accompanying posture (tail position) should definitely be taken into account for reliable further interpretation of the performed behaviour.

None of the other behaviours investigated in the present study were found to be useful as status indicators, since they were highly bidirectional or did not have sufficient coverage or did not show significant linearity. Although aggressive behaviours like *stare*, *growl*, *show teeth* and *bite* are labelled as dominant behaviour by some authors [37,38], in the current study none of these behaviours met the criteria for suitable rank indicator, especially because of insufficient unidirectionality. This compares to the outcomes of the more detailed analyses of aggressive wolf–wolf interactions, where no link between bared teeth and rank position could be revealed unless the retraction of the lips was taken into account [58]. Moreover, in our study aggressive behaviours have been observed to occur simultaneously with lowered postures, which contradicts their interpretation as dominant behaviours.

### Steepness and dominance style

Alongside linearity, as a quantitative measure to characterize a dominance hierarchy, we used steepness [42]. A steep rank order indicates large asymmetries between individuals. This in turn is a characteristic of a despotic, less tolerable society. In a more tolerable society, smaller asymmetries will be found between individuals, and consequently a less steep rank order should be found. In macaque species, steepness has been used, along with asymmetry, intensity of aggression and the use of status signals, to distinguish four types of dominance styles [13]: (1) despotic, (2) tolerant, (3) relaxed, and (4) egalitarian. Using steepness as an indicator and following these labels, the dominance style in our group can be best indicated as ‘tolerant’ for the following reasons: (a) the degree of steepness itself (0.79 when based on *Pij*; 0.67 when based on *Dij*) (0.79 when based on Pij; 0.67 when based on Pij)) fits within the variation of steepness found for styles 2 and 3 in macaque species [59]; (b) large dyadic asymmetries in postural displays (specifically *LoP* and *high posture*) were reinforced with mild to moderate aggression (e.g. *stare*, *pilo-erection*, *growl*, *show teeth*, *snap*); (c) both dominant and subordinate dogs signaled their roles using formal status signals; (d) many relationships were formalized, while some relationships (especially those in the middle ranking group and in the lowest ranking group) were unresolved, but they were not avoiding each other (see approach matrix in the S1 Appendix) and (e) aggressive behaviours of low intensity had high bidirectional exchange. The steepness of the hierarchy, research on macaque species suggests, covaries with dominance or social style [60]. High steepness indicates strong asymmetries between the individuals and large absolute rank distances between high and low ranking individuals. Investigation of the relations between steepness and dyadic aggression characteristics such as bidirectional aggression, counter-aggression, aggression intensity and conflict resolution efficacy or reconciliation, therefore, should be the focus of future research in dog groups.

### Concept of dominance as intervening variable

To assess the usefulness of the concept of dominance as intervening variable, we determined the agreement between distributions of different postural and behavioural variables. The variables *lowering of posture*, *high posture* and *body tail wag*, all status indicators at group level, were found to show a high covariation. This finding underlines existing asymmetries between individuals and shows dominance to be a useful intervening variable in explaining a social organisation. Also, this finding corresponds closely with wolf behaviours (*low*, *active submission* and *high*) in the dominance-subordination domain as identified in the Burger’s zoo wolves ([9], p 236). Aggressive behaviours conventionally associated with the concept of dominance, like *growl*, *show teeth* and *snap*, clearly stand apart from the dominance and subordination status signals in our dog group as discussed above. This confirms the findings in the Burger’s zoo wolf study, that *threat/assault* clearly stands apart from the dominance-subordination domain. In conclusion, we showed that the concept of dominance, as shown in captive wolves and free-ranging dogs, is applicable in our group of dogs and is reflected foremost by postural displays, and not by aggressive displays.

## Conclusions

This systematic study on the applicability of the concept of dominance revealed formal dominance to be applicable to domestic dogs. As a direct application of our findings, we could characterize the dominance style in this study group of dogs as ‘tolerant’. These findings are useful in correctly interpreting the relationship between dogs at the dyadic level regarding status as well as for the position in the group (rank order). The method described herein could be applied to determine breed-related differences, and moreover these findings could be helpful in correctly diagnosing the status in dog-dog or dog-human relationships in case of behavioural problems, based on formal signals and not aggression.

The question can be raised whether the tolerant dominance style found in our group can be generalized to other dog groups. In macaque species, for example, not only are there variations in dominance style within (female *vs* males) and between groups of the same species, but also between species [60]. As dog breeds vary highly in morphology, aggressive tendencies and temperament or personality [55,61,62,63] we expect to see variation in dominance style that will be highly dependent on the breed composition of the group. Bold breeds (e.g. Rottweilers, Malinois) might show a more despotic dominance style, whereas, shy breeds (e.g. Cavalier King Charles Spaniels, Labrador retrievers) might exhibit a more relaxed or even an egalitarian dominance style. In mixed breed groups the dominance style of the highest ranking male / female would reasonably be expected to heavily influence the steepness of the hierarchy. In view of this potentially great variation regarding behavioural tendencies both between breeds and within breeds [64], it is to be expected that all types of dominance styles will be found in dogs. Thus while the characterization of our study group cannot be generalized to other dog groups, the method used can be, and future research should reveal the extent to which different dominance styles are present in group housed domestic dogs of different breeds and breed types.

## Supporting Information

S1 Appendix Three dyadic dominance related matrices ordered by the normalized David’s score; (a-c): *LoP*, *high posture* and *body tail wag*. NDS = Normalized David’s score based on *P*_*ij*_. The individuals in the fourth matrix (d), *approach*, are ordered in *LoP* rank order, for comparison purposes.
(DOCX)

S1 Fig Cluster analysis (average linkage method) of *Lowering of posture* and *High* posture and 11 behaviours. The Pearson correlation between NormDS values are used as similarity measure. The correlations are rescaled to a distance measure which varies between 0 and 25, such that the ratios of these distances are identical to the ratios of the original correlations (or, to the ratios of the average correlations between pairs of behaviours in different clusters).
(PNG)

S1 File Comparison cluster analysis using NormDS based on *Pij* with cluster analysis based on *Dij*. (PDF)
