# Supplementary material for: Dominance in Domestic Dogs: A Quantitative Analysis of Its Behavioural Measures
Source: PLoS One. 2015 Aug 26;10(8):e0133978. doi: 10.1371/journal.pone.0133978 (PMC4556277; doi:10.1371/journal.pone.0133978)
Supplement: S1 Appendix — Three dyadic dominance related matrices ordered by the normalized David’s score; (a-c): LoP, high posture and body tail wag. NDS = Normalized David’s score based on P ij. The individuals in the fourth matrix (d), approach, are ordered in LoP rank order, for comparison purposes. (DOCX) [file pone.0133978.s001.docx]

**S1 Appendix.** Three dyadic dominance related matrices ordered by the normalized David’s score; (a-c): *LoP*, *high posture* and *body tail wag*. NDS = Normalized David’s score based on *P_ij_*. The individuals in the fourth matrix (d), *approach*, are ordered in *LoP* rank order, for comparison purposes.

1. LoP (row individuals are the receivers)

|  | **W** | | **P** | | **T** | | **V** | | **I** | **U** | | **B** | | **K** | | **Z** | **S** | | **Total** | | **NDS** | | |  |  |
| --- | --- | --- | --- | --- | --- | --- | --- | --- | --- | --- | --- | --- | --- | --- | --- | --- | --- | --- | --- | --- | --- | --- | --- | --- | --- |
| **W** | * | | 42 | | 1 | | 1 | | 2 | 5 | | 3 | | 10 | | 7 | 15 | | 86 | | 8.05 | | |  |  |
| **P** | 2 | | * | | 85 | | 32 | | 6 | 71 | | 45 | | 31 | | 12 | 40 | | 324 | | 7.65 | | |  |  |
| **T** | 1 | | 0 | | * | | 7 | | 14 | 12 | | 5 | | 11 | | 2 | 16 | | 68 | | 6.89 | | |  |  |
| **V** | 0 | | 0 | | 0 | | * | | 1 | 1 | | 0 | | 8 | | 1 | 14 | | 25 | | 4.80 | | |  |  |
| **I** | 0 | | 0 | | 1 | | 1 | | * | 1 | | 1 | | 9 | | 2 | 11 | | 26 | | 4.77 | | |  |  |
| **U** | 0 | | 0 | | 1 | | 0 | | 1 | * | | 0 | | 2 | | 4 | 6 | | 14 | | 3.98 | | |  |  |
| **B** | 0 | | 0 | | 0 | | 0 | | 0 | 0 | | * | | 1 | | 2 | 2 | | 5 | | 3.79 | | |  |  |
| **K** | 0 | | 0 | | 1 | | 0 | | 0 | 0 | | 0 | | * | | 0 | 3 | | 4 | | 2.08 | | |  |  |
| **Z** | 0 | | 0 | | 0 | | 0 | | 0 | 0 | | 0 | | 0 | | * | 0 | | 0 | | 1.89 | | |  |  |
| **S** | 0 | | 0 | | 0 | | 0 | | 0 | 0 | | 0 | | 0 | | 0 | * | | 0 | | 1.10 | | |  |  |
| **Total** | 3 | | 42 | | 89 | | 41 | | 24 | 90 | | 54 | | 72 | | 30 | 107 | | 552 | |  | | |  |  |
|  | |  | |  | |  | |  | | |  | |  | |  | | |  | |  | |  |  | |  |

1. High posture (row individuals are the displayers)

|  | **W** | **P** | **U** | **T** | **I** | **B** | **Z** | **V** | **K** | **S** | **Total** | **NDS** |
| --- | --- | --- | --- | --- | --- | --- | --- | --- | --- | --- | --- | --- |
| **W** | * | 13 | 6 | 6 | 11 | 3 | 8 | 10 | 7 | 20 | 84 | 7.68 |
| **P** | 0 | * | 8 | 12 | 2 | 6 | 3 | 6 | 6 | 9 | 52 | 7.30 |
| **U** | 3 | 0 | * | 2 | 8 | 7 | 6 | 11 | 18 | 16 | 71 | 6.09 |
| **T** | 2 | 0 | 1 | * | 0 | 1 | 2 | 3 | 1 | 2 | 12 | 4.86 |
| **I** | 1 | 0 | 1 | 0 | * | 0 | 2 | 4 | 6 | 6 | 20 | 4.66 |
| **B** | 0 | 0 | 1 | 1 | 0 | * | 0 | 1 | 1 | 1 | 5 | 4.52 |
| **Z** | 0 | 0 | 0 | 1 | 0 | 0 | * | 0 | 0 | 0 | 1 | 3.33 |
| **V** | 0 | 0 | 0 | 0 | 0 | 0 | 0 | * | 0 | 1 | 1 | 2.72 |
| **K** | 0 | 0 | 0 | 0 | 0 | 0 | 0 | 0 | * | 1 | 1 | 2.22 |
| **S** | 0 | 0 | 1 | 0 | 0 | 0 | 0 | 0 | 1 | * | 2 | 1.63 |
| **Total** | 6 | 13 | 18 | 22 | 21 | 17 | 21 | 35 | 40 | 56 | 249 |  |
|  |  |  |  |  |  |  |  |  |  |  |  |  |

1. Body tail wag (row individuals are the receivers)

|  | **W** | **P** | **I** | **T** | **U** | **V** | **B** | **K** | **Z** | **S** | **Total** | **NDS** |
| --- | --- | --- | --- | --- | --- | --- | --- | --- | --- | --- | --- | --- |
| **W** | * | 5 | 2 | 0 | 10 | 2 | 6 | 6 | 7 | 1 | 39 | 7.42 |
| **P** | 0 | * | 5 | 45 | 78 | 25 | 53 | 17 | 5 | 3 | 231 | 6.87 |
| **I** | 0 | 2 | * | 1 | 0 | 1 | 1 | 0 | 1 | 1 | 7 | 5.61 |
| **T** | 0 | 0 | 0 | * | 4 | 4 | 1 | 5 | 1 | 1 | 16 | 5.28 |
| **U** | 0 | 0 | 0 | 0 | * | 1 | 1 | 5 | 3 | 1 | 11 | 4.45 |
| **V** | 0 | 0 | 1 | 1 | 0 | * | 0 | 0 | 0 | 0 | 2 | 3.74 |
| **B** | 0 | 0 | 0 | 0 | 0 | 0 | * | 4 | 4 | 1 | 9 | 3.43 |
| **K** | 0 | 0 | 0 | 0 | 1 | 0 | 0 | * | 0 | 0 | 1 | 2.98 |
| **Z** | 0 | 0 | 0 | 0 | 1 | 0 | 0 | 0 | * | 0 | 1 | 2.74 |
| **S** | 0 | 0 | 0 | 0 | 0 | 0 | 0 | 0 | 0 | * | 0 | 2.49 |
| **Total** | 0 | 7 | 8 | 47 | 94 | 62 | 33 | 37 | 21 | 8 | 317 |  |

1. Approach (row individuals are approachers)

|  | **W** | **P** | **T** | **V** | **I** | **U** | **B** | **K** | **Z** | **S** | **Total** |
| --- | --- | --- | --- | --- | --- | --- | --- | --- | --- | --- | --- |
| **W** | * | 21 | 8 | 17 | 17 | 16 | 12 | 32 | 27 | 34 | 184 |
| **P** | 41 | * | 17 | 20 | 5 | 17 | 4 | 11 | 10 | 13 | 138 |
| **T** | 51 | 63 | * | 39 | 33 | 80 | 33 | 22 | 8 | 29 | 358 |
| **V** | 24 | 35 | 17 | * | 22 | 21 | 12 | 26 | 10 | 34 | 201 |
| **I** | 53 | 32 | 5 | 30 | * | 12 | 14 | 42 | 9 | 47 | 244 |
| **U** | 29 | 25 | 20 | 9 | 17 | * | 25 | 9 | 6 | 12 | 152 |
| **B** | 14 | 18 | 31 | 9 | 8 | 25 | * | 3 | 4 | 5 | 117 |
| **K** | 13 | 15 | 25 | 14 | 10 | 15 | 9 | * | 10 | 41 | 152 |
| **Z** | 9 | 4 | 4 | 15 | 4 | 7 | 7 | 8 | * | 2 | 60 |
| **S** | 30 | 10 | 9 | 18 | 9 | 21 | 7 | 33 | 1 | * | 138 |
| **Total** | 264 | 223 | 136 | 171 | 125 | 214 | 123 | 186 | 85 | 217 | 1744 |
