# Supplementary figures and images for: Dominance in Domestic Dogs: A Quantitative Analysis of Its Behavioural Measures
Source: PLoS One. 2015 Aug 26;10(8):e0133978. doi: 10.1371/journal.pone.0133978 (PMC4556277; doi:10.1371/journal.pone.0133978)

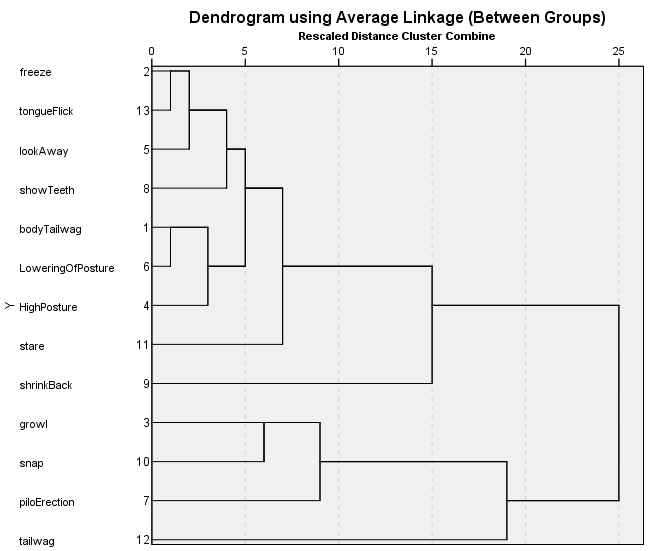

Supplement: S1 Fig — The Pearson correlation between NormDS values are used as similarity measure. The correlations are rescaled to a distance measure which varies between 0 and 25, such that the ratios of these distances are identical to the ratios of the original correlations (or, to the ratios of the average correlations between pairs of behaviours in different clusters). (PNG) [file pone.0133978.s002.png]
