# Supplementary material for: Dominance in Domestic Dogs: A Quantitative Analysis of Its Behavioural Measures
Source: PLoS One. 2015 Aug 26;10(8):e0133978. doi: 10.1371/journal.pone.0133978 (PMC4556277; doi:10.1371/journal.pone.0133978)
Supplement: S1 File — (PDF) [file pone.0133978.s003.pdf]

S1 File. Comparison cluster analysis using NormDS based on *Pij* with cluster analysis based on *Dij*.

We performed the same type of cluster analysis using NormDS based on D_{ij}, which resulted in virtually the same cluster dendrogram. D_{ij} is a measure of dyadic dominance from individual *i* to individual *j*, similar to P_{ij} but corrected for chance and interaction frequency. Specifically, $P_{ij} = x_{ij}/(x_{ij}+x_{ji})$; $D_{ij} = (x_{ij} + 0.5)/(x_{ij}+x_{ji} +1)$, where x_{ij} is number of interactions from individual *i* to individual *j* [42]. It has been shown that D_{ij}-based and P_{ij}-based rank orders are very comparable to each other [51]. Interestingly, when we compared the NormDS-$\mathrm{P_{ij}}$ and NormDS-$\mathrm{D_{ij}}$ rank orders of *LoP* with each other, we found for the two highest dogs a different order. Based on P_{ij} we found NormDS of dog W = 8.05 and NormDS of dog P = 7.65; based on D_{ij} however, we found NormDS of dog W = 7.36 and NormDS of dog P = 7.49. So, when using the overall dominance success score based on P_{ij} dog W scores higher than dog P, whereas with D_{ij} dog P scores higher than dog W. From the viewpoint of 'overall dominance success score' this last outcome (dog P has a higher score than dog W), is understandable, since dog P received much more *LoP*'s from all other dogs than dog W did (see the *LoP* matrix in the Appendix). However, from the viewpoint of 'dyadic dominance relationships' it is not. Looking at the dyadic interactions between dogs W and P, clearly P is subordinate to W, since P lowered its posture 42 times towards W, whereas W lowered its posture only 2 times towards P. So, in this particular case the overall dominance success score measure 'NormDS based on $\mathrm{D_{ij}}$' gave a result that contradicts the dyadic dominance relationships outcome. Therefore, we decided to present the results of NormDS based on P_{ij} rather than those based on D_{ij}.
